# Terahertz and Millimeter Wave Sensing and Applications

**DOI:** 10.3390/s22249693

**Published:** 2022-12-10

**Authors:** Maris Bauer, Fabian Friederich

**Affiliations:** Department of Materials Characterization and Testing, Fraunhofer Institute for Industrial Mathematics ITWM, DE-67663 Kaiserslautern, Germany

The field of terahertz and millimeter wave science and technology has evolved in recent years into an area attracting a lot of attention from all sides of science, industry, and the public. The foremost reason for this increased attention has been the development of powerful sources and sensitive detectors for this particular radiation band of the electromagnetic spectrum. The term *terahertz* is commonly defined to address electromagnetic waves with frequencies from around 0.1 to 10 THz and respective wavelengths of several millimeters to tens of micrometers. At its lower-frequency end, the terahertz region crosses over with so-called *millimeter waves*, with an upper boundary around 300 GHz, and reaching down, depending on the definition, to several tens of GHz. The research and development of terahertz components and system concepts has enabled the application of terahertz waves in countless areas reaching from fundamental scientific research, e.g., of carrier dynamic processes in semiconductors or astrophysical observation of gas clouds, to real-world application scenarios, e.g., non-destructive material testing and quality control or high-speed wireless communication. A large number of review articles exist, which give good overview of the history of terahertz science and the various areas where terahertz technology is found today. Some of them—without any claim of completeness—are References [1,2,3,4,5,6] and the many references therein.

The early years of terahertz science were long dominated by the search for methods of direct terahertz generation with significant power levels—other than radiation generation from broadband emission sources—and, at the same time, the development of radiation detectors and measurement concepts for this frequency range. Naturally, approaches situated on the lower end of the millimeter wave and terahertz spectra were mostly based on the frequency extensions of widespread radio and high frequency technologies—commonly summarized as electronic millimeter and terahertz technologies. On the high-frequency end, approaches extending established technologies from the infrared into the terahertz regime are mostly based on opto-electronic system concepts and are commonly summarized as optical terahertz technologies. The particular challenges of implementing such established technologies in the millimeter and terahertz frequency range was long associated with the term terahertz gap, which emphasized the lack of application-ready technologies in this spectral region. Nevertheless, the continuous research in the field has led to great improvements on both the source and detector side, and finally, many terahertz technologies have reached a level of maturity which allows them to be applied in real-world industrial contexts and beyond.

This Special Issue of MDPI *Sensors* is intended to bring together some recent publications related to the application of millimeter and/or terahertz technology to specific application-related implementations in various fields. It covers a number of particular application examples employing terahertz radiation [7,8,9,10,11,12], presents methodological approaches for measurements techniques and concepts [13,14,15,16,17,18,19], as well as some contributions dedicated to recent achievements from the technology side of source and detector development [20,21,22].

A nice example showing how various conventional radar and signal processing techniques can be exploited in the millimeter and terahertz regime, as the respective technical system components become available, is presented by Wagner et al. [7]. In their contribution, the authors demonstrate the use of a millimeter wave frequency-modulated continuous wave (FMCW) radar for the detection of drilling intrusion in sensitive transport containers. Another such radar technique is employed in the contribution by Batra et al., namely, synthetic aperture radar (SAR) [8]. The authors present machine learning-supported object classification by feature extraction from images obtained with an indoor terahertz SAR system operating at frequencies of 325 to 500 GHz. The identification of humans is the subject of a publication by Alkasimi et al., where deep transfer learning is applied to millimeter wave waveforms of the heart sound of walking individuals in 77 GHz measurements at standoff distances [9]. The publication by Zarrinkhat et al. demonstrates the application of terahertz imaging in the field of medical applications [10], in particular the assessment of special calibration requirements in the investigation of corneal thickness and water content towards an early diagnosis technique for corneal dystrophies.

Another established field where terahertz radiation is widely employed is spectroscopic material identification. Many characteristic molecular vibrational and rotational (in gases) absorption bands are located in the terahertz spectral region. Mostly time-domain spectroscopy (TDS) systems are currently being employed for spectroscopic measurements. In their contribution to this Special Issue, Im et al. demonstrate experiments towards a highly relevant topic today, namely, the detection of microplastics, in their case mixed into table salts [11]. Effective medium theory is applied to TDS signals between 0.1 and 0.5 THz to identify different volume ratios of HDPE and salt mixtures in powder form. Zhai et al. show in their publication that terahertz TDS can also be useful to support the automated handling of paper documents [12]. By extracting page counts of paper stacks as well as detecting sheet thickness, moisture content, and possible foreign objects, the technique could be employed in, e.g., paper feeders of document scanners.

Several techniques for the analysis of terahertz TDS waveforms can be used to extract valuable information with various application scenarios in mind. Khani et al. investigate in their publication the advantages of maximum overlap discrete wavelet transform (MODWT) over conventional DWT for the extraction of spectral features at the example of α-lactose monohydrate pellets [13]. In another contribution, Ren at al. present a compressed sensing approach to exploit the dual sparsity of terahertz TDS data in the wavelet and gradient domains for the reconstruction of terahertz images [14]. The authors evaluate their method by performing TDS transmission imaging of wheat flour pellets and wheat seeds.

A number of contributions to this Special Issue presented innovative concepts on the system side and measurement techniques. For example, the design of a W-Band, horn antenna-integrated photonic receiver on the basis of a electro-optical whispering gallery mode resonator is proposed by Strekalov et al. [15] for the use of cloud and precipitation radar. Another radar approach is contributed by Konno et al. presenting discrete Fourier transform-based distance measurements in an all-semiconductor-based radar system [16]. The practical issues when using solid immersion lenses in terahertz imaging and methods of interference pattern removal are the content of a paper by Choi et al. [17]. An interesting system concept based on the use of a monolithic mode-locked laser diode to generate terahertz radiation in photomixers for Mach–Zehnder-type interferometric thickness measurements is presented by Kolpatzeck et al. [18]. The authors report an accuracy of few tens of micrometers of layer thicknesses with their measurement setup. In a work by Kim et al. [19], an approach for plasma diagnostics by measuring the electron density in the so-called crossing frequency method with the help of microwave probes is presented.

On the device level, Paz-Martínez et al. investigated the performance of field-effect transistors as terahertz detectors based on GaN HEMTs in dependence of temperature and gate-length variation [20]. The same device type of field-effect transistor-based terahertz detector with integrated receiving antennas (TeraFET) but fabricated using commercial foundry-based silicon CMOS technology is presented by Ikamas et al. [21], where the detectors are combined in a free-space transmission system with also silicon CMOS-based emitters at 250 GHz. A possible application discussed in the paper is the emerging field of terahertz communication. Finally, we were happy to include in this Special Issue a perspective article, in which the authors Shur, Aizin, Otsuji, and Ryzhii focus further on this topic and present their opinion on the future of TeraFETs and in general plasmonic terahertz semiconductor devices for 6G communication [22].

We thank all the authors of the above papers for making this Special Issue possible with their submissions. We believe that their contributions present a good insight into recent developments in the field of terahertz and millimeter wave applications and system concepts, emphasizing that terahertz and millimeter wave technologies have finally reached a level of maturity to be applied in countless fields of both scientific and industrial contexts.

## References

[1] Siegel P.H. (2003). THz Technology: An Overview. Int. J. High Speed Electron. Syst..

[2] Lewis R.A. (2014). A review of terahertz sources. J. Phys. D Appl. Phys..

[3] Dhillon S.S., Vitiello M.S., Linfield E.H., Davies A.G., Hoffmann M.C., Booske J., Paoloni C., Gensch M., Weightman P., Williams G.P. (2017). The 2017 terahertz science and technology roadmap. J. Phys. D Appl. Phys..

[4] Mittleman D.M. (2017). Perspective: Terahertz science and technology. J. Appl. Phys..

[5] Naftaly M., Vieweg N., Deninger A. (2019). Industrial Applications of Terahertz Sensing: State of Play. Sensors.

[6] Hillger P., Grzyb J., Jain R., Pfeiffer U.R. (2019). Terahertz Imaging and Sensing Applications With Silicon-Based Technologies. IEEE Trans. Terahertz Sci. Technol..

[7] Wagner S., Alkasimi A., Pham A.V. (2022). Detecting the Presence of Intrusive Drilling in Secure Transport Containers Using Non-Contact Millimeter-Wave Radar. Sensors.

[8] Batra A., Sheikh F., Khaliel M., Wiemeler M., Göhringer D., Kaiser T. (2022). Object Recognition in High-Resolution Indoor THz SAR Mapped Environment. Sensors.

[9] Alkasimi A., Shepard T., Wagner S., Pancrazio S., Pham A.V., Gardner C., Funsten B. (2022). Dual-Biometric Human Identification Using Radar Deep Transfer Learning. Sensors.

[10] Zarrinkhat F., Baggio M., Lamberg J., Tamminen A., Nefedova I., Ala-Laurinaho J., Khaled E.E.M., Rius J.M., Romeu J., Taylor Z. (2022). Calibration Alignment Sensitivity in Corneal Terahertz Imaging. Sensors.

[11] Im J., Goo T., Kim J., Choi S., Hong S.J., Bahk Y.M. (2021). Detection of Microplastic in Salts Using Terahertz Time-Domain Spectroscopy. Sensors.

[12] Zhai M., Locquet A., Citrin D.S. (2021). Terahertz Imaging for Paper Handling of Legacy Documents. Sensors.

[13] Khani M.E., Arbab M.H. (2022). Translation-Invariant Zero-Phase Wavelet Methods for Feature Extraction in Terahertz Time-Domain Spectroscopy. Sensors.

[14] Ren X., Jiang Y. (2021). Spatial Domain Terahertz Image Reconstruction Based on Dual Sparsity Constraints. Sensors.

[15] Strekalov D., Majurec N., Matsko A., Ilchenko V., Tanelli S., Ahmed R. (2022). W-Band Photonic Receiver for Compact Cloud Radars. Sensors.

[16] Konno H., Dobroiu A., Suzuki S., Asada M., Ito H. (2021). Discrete Fourier Transform Radar in the Terahertz-Wave Range Based on a Resonant-Tunneling-Diode Oscillator. Sensors.

[17] Choi D.H., Shin J.H., Lee I.M., Park K.H. (2021). Investigations on Practical Issues in Solid Immersion Lens Based Sub-Wavelength Terahertz Imaging Technique: System Stability Verification and Interference Pattern Removal. Sensors.

[18] Kolpatzeck K., Liu X., Häring L., Balzer J.C., Czylwik A. (2021). Ultra-High Repetition Rate Terahertz Time-Domain Spectroscopy for Micrometer Layer Thickness Measurement. Sensors.

[19] Kim S.j., Lee J.j., Lee Y.s., Cho C.h., You S.j. (2022). Crossing Frequency Method Applicable to Intermediate Pressure Plasma Diagnostics Using the Cutoff Probe. Sensors.

[20] Paz-Martínez G., Íñiguez-de-la Torre I., Sánchez-Martín H., Novoa-López J.A., Hoel V., Cordier Y., Mateos J., González T. (2022). Temperature and Gate-Length Dependence of Subthreshold RF Detection in GaN HEMTs. Sensors.

[21] Ikamas K., But D.B., Cesiul A., Kołaciński C., Lisauskas T., Knap W., Lisauskas A. (2021). All-Electronic Emitter-Detector Pairs for 250 GHz in Silicon. Sensors.

[22] Shur M., Aizin G., Otsuji T., Ryzhii V. (2021). Plasmonic Field-Effect Transistors (TeraFETs) for 6G Communications. Sensors.

